# Data Integration from Heterogeneous Control Levels for the Purposes of Analysis within Industry 4.0 Concept

**DOI:** 10.3390/s22249860

**Published:** 2022-12-15

**Authors:** Tibor Horak, Peter Strelec, Michal Kebisek, Pavol Tanuska, Andrea Vaclavova

**Affiliations:** Institute of Applied Informatics, Automation and Mechatronics, Faculty of Materials Science and Technology in Trnava, Slovak University of Technology in Bratislava, 91724 Trnava, Slovakia

**Keywords:** integration, big data, data analysis, manufacturing execution system, production line

## Abstract

Small- and medium-sized manufacturing companies must adapt their production processes more quickly. The speed with which enterprises can apply a change in the context of data integration and historicization affects their business. This article presents the possibilities of implementing the integration of control processes using modern technologies that will enable the adaptation of production lines. Integration using an object-oriented approach is suitable for complex tasks. Another approach is data integration using the entity referred to as tagging (TAG). Tagging is essential to apply for fast adaptation and modification of the production process. The advantage is identification, easier modification, and generation of data structures where basic entities include attributes, topics, personalization, locale, and APIs. This research proposes a model for integrating manufacturing enterprise data from heterogeneous levels of management. As a result, the model and the design procedure for data integrating production lines can efficiently adapt production changes.

## 1. Introduction

Data integration is a commonly used term in industry that refers to the requirement to combine data from multiple separate enterprise systems into a single data entity. In the context of information technology and industry, integration refers to the result of a process that brings together different, often disparate, subsystems so that the data contained in each becomes part of a larger, more complex system [1]. 

If needed, this system can share data quickly and easily. This often requires companies to create a customized architecture or application structure to combine new or existing hardware, software, and other communication means [2]. 

The unified data unit thus created is stored on a data storage device. The aim is to analyze the data generated using the manufacturing process for streamlining the manufacturing process [3]. 

Data from transactional systems, relational databases, and other sources are stored in the data warehouse, usually at regular intervals. The repository needs to be appropriately selected according to the size and type of data that will be retrieved from the manufacturing process [4].

Integration can be divided into vertical and horizontal in terms of production process control. Horizontal integration refers to the integration of data from the same level of management, as opposed to vertical integration, which is the integration between different levels of management [5].

Horizontal integration is described as one in which a corporation, to create an efficient production system, should both cooperate and compete with corporations that have similar characteristics. Material, financial control, and knowledge can easily be linked in all these corporations. Therefore, new control systems and business models may emerge [6]. 

The vertical integration introduces the idea of a factory that has various information and physical subsystems, such as production control, actuator and sensor, value, and enterprise planning. The vertical integration of sensor and actuator signals along the different levels of the enterprise resource planning (ERP) level provides high flexibility and simplicity in the configuration of production lines. From this integration, highly intelligent machines form an automated controlled system that can be automatically reconfigured according to different product types. The large amount of data collected and processed allows the production system to be transparent [7].

The integration of end-to-end engineering in the chain of activities throughout the product-oriented value creation process includes aspects such as customer requirements expression, product development and design, recycling, production engineering, manufacturing services, production planning, and maintenance. From end-to-end integration, each phase can be reused for the same product model. The impact of product design on services and production can be predicted using a software tool in the chain to ensure that products are customizable [8].

The aim of data integration is to link existing elements to enable easier production management. The companies in which data integration has been carried out have been analyzed. Enterprises that have not implemented integration for some reason find it difficult to identify the potential benefits that this area brings. For this purpose, an overview of enterprises in which data integration has been implemented using a manufacturing execution system (MES) has been created [9]. 

The current possibilities of integrating industrial internet of Things (IIoT) devices for streamlining production processes in small- and medium-sized enterprises are discussed on the basis of the analysis of production data that resulted from the implemented integration [10].

Data integration is intended to provide basic access to data. It is a method for blending data from a hybrid pool of data, converting it into meaningful data, and providing results and insights to the user or client according to their business needs for effective purposes. It is a method that combines technical and business operations to extract data from different sources through a reliable and accurate analysis of the right data [11].

### 1.1. Survey on the Deployment of Integration and Industry 4.0 Elements

**Figure 1:**
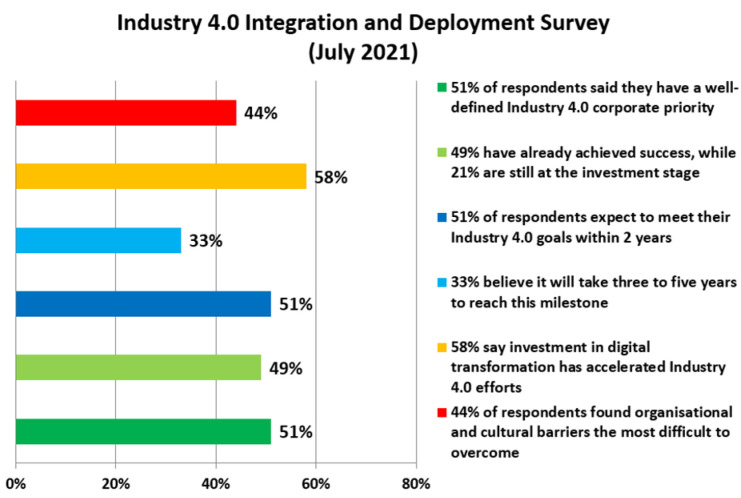
Integration deployment survey.

From the survey shown in Figure 1, which was conducted in 2021, an overview of enterprise systems’ integration deployments is produced with the goal of data integration and Industry 4.0 deployments. The research aimed to gather data on practical experience with Industry 4.0. A total of 216 practitioner respondents answered the questions and the survey confirmed continued growth and potential benefits for customers [12].

### 1.2. Motivation for this Article

When integrating the data, there are various problems encountered that need to be considered in the solution. One of the biggest problems is the reliability of the solution. It is imperative that the data integration and integration of all management layers provide a reliable solution, because if any part fails, the entire production can be shut downthe production line can come to a standstill, which represents a potential financial loss [13].

Another issue is safety. Since, in the case of integration of production processes, enterprises will receive a huge amount of data, it is necessary to protect them from possible hacker attacks [14]. 

The financial aspect must be considered from several angles. Firstly, it must be verified whether data integration is necessary at all and whether it is worth investing financial resources usually considerable in its implementation. Naturally, after deploying integration, manufacturing companies will gain much “new” data and thus potentially increase production efficiency, which is clearly a benefit, but it is necessary to decide in what time frame the return on investment is realistic and, thus, whether it is profitable at all [15].

Additional funds need to be spent on the training staff to be able to operate and work with the newly integrated systems, and the fact that, during these training sessions, the production staff will be absent needs to be kept in mind [16].

The network infrastructure also needs to be thought about, as a huge amount of data will be acquired by the integration. It is necessary to design the network infrastructure of the information systems so that it is reliable and sufficiently oversized, which is represented, for example, by sufficient storage capacity and reliable infrastructure for the preparation of the integration of information systems or lower-level communications [17,18].

All these factors led to the realization of this paper, since the consideration of these aspects is scarcely available in the literature. Enterprises that have implemented system integration guard their know-how. This article presents a proposal of two models from which a general proposal of the process of realizing the integration of manufacturing systems will emerge. The application of the presented solution will help manufacturing enterprises in deploying integration.

## 2. Materials and Methods

Manufacturing execution systems, commonly referred to as MESs, are implemented in many manufacturing companies as effective interfaces between process-level control systems, typically supervisory control and data acquisition (SCADA), and enterprise information systems, which include enterprise resource planning (ERP)-type systems used to plan and manage corporate resources [19]. 

Thus, MES links the two different and independent levels to form coherent information and control system that enable the easy exchange of information relevant to decision making, planning, control, analysis, and evaluation of production across the enterprise [20]. 

For integration, it is necessary to collect data. The methods for data preparation according to structural type are structured and unstructured [21].

### 2.1. Structured and Unstructured Data

Data exist in different forms and sizes, but most of them can be presented as structured or unstructured data [22].

Structured data are a type of data that are well organized and precisely formatted. These data exist in relational database management system (RDBMS) format, and included structured forms such as eXtensible Markup Language (XML) or JavaScript Object Notation (JSON)—which means that the information is stored in tables with linked rows and columns. In this way, the structured data are neatly organized and recorded so that they can be easily found, processed, and analyzed [23]. 

As long as the data fit into the structure of the RDBMS, specific information and extraction of relationships between parts of it could be easily found. Such data can only be used for their intended purpose. Moreover, structured data usually do not require much storage space. Thus, for analysis purposes, classic data warehouses can be used. These are commonly used by companies for analysis and report generation [24].

Unstructured data are information in different forms that do not relate to common data models and, therefore, are usually not suitable for a conventional relational database. Therefore, they are more difficult to analyze and are not easily retrievable; for this reason, such storage has not been useful for companies until recent years [25].

With the emergence of alternative platforms for storing and managing such data, they are becoming increasingly common in IT systems and are used by organizations in various business intelligence and analytics applications [26].

### 2.2. Processing of Unstructured Data

The most common method is the transformation of unstructured data into structured data using processes such as normalization, categorization, and syntactic analysis. This method is often used for all technology platforms. There are different methods of transformation into structured data. Commonly used methods are:

- Normalization—data that are expressed in different ways are reduced to so-called canonical data (to a standardized format).
- Categorization—distinguishing data from different sources into so-called categories using tags. It is the grouping of objects according to certain properties.
- Syntactic parsing (also called parsing)—analyzing a sequence of formal elements to determine grammatical structure versus formal grammar. Thus, it is the transformation of text into a concrete structure.
- Content analysis. The aim of this method is to identify key themes and concepts that occur in the text using various statistical methods.
- Contextual analysis. Contextual analysis allows us to automatically evaluate the occurrence of defined themes in the full texts of the processed documents. It is a quantitative type of data analysis used to analyze unstructured data.
- Sentiment analysis. This method detects the attitude of the author of a text towards a given topic and then classifies it into one of three groups: positive, negative, and neutral.
- Structure mining. This is a special method of extracting semi-structured data, most often XML files. It is used in cases when it is necessary to process both the content of a document and its more detailed specifications (metadata, etc.). To extract this data, the so-called XPath computer language is used, which allows one to retrieve certain data in the structure of XML documents [27,28].

### 2.3. Integration Types

Companies are now implementing several data integration techniques. These are addressed by a number of techniques, and an overview of these is provided here:

- Manual integration or a common user interface—provides users with access to all source systems or web interfaces.
- Application-based integration—used only for a finite number of applications. Middleware data integration—helps transfer logic from one application to a new middleware layer.
- Unified data access or virtual integration—defines a set of views that give users access to a single view of the data.
- Shared data storage or physical data integration—contains a copy of the data from the source and stores and manages it independently of the original system [29].

### 2.4. Data Integration

Data consolidation essentially combines data from several individual systems to create a single data warehouse. The goal of data consolidation is to achieve a reduction in the number of data storage locations, which is supported by extract transform load (ETL), an extract, transform, and load technology. ETL extracts data from the data stores, transfers it into a readable format, and then transfers it to another data warehouse [30].

- Data dissemination. By using the application, data are duplicated from one place to another. The duplication can be accomplished in two ways between the source and the client. Data dissemination is supported by enterprise data replication and enterprise application integration. Enterprise application integration (EAI) manages applications sharing messages and is usually performed in real time. An event data recorder (EDR) transfers huge amounts of data between databases, which are used to query and distribute data sharing between source and servers [31].
- Data virtualization. Virtualization manages the interface and offers unique data from separate sources with different data models. Data virtualization interprets and extracts data from any source without a single point of contact [32].
- Data federation. This is a theoretical form of data virtualization that uses virtual databases to create a generic data model for hybrid data from different systems. Data are collected from different sources and made available in a single view. Data abstraction is intended to provide a discrete view of the data from a hybrid source by integrating enterprise information. Data can be analyzed in a trend-driven manner using multiple applications. Data consolidation is expensive because of advanced security and compliance features [33,34].

Data integration improves the customer experience by offering an instant service. It provides a controlled flow of data that streamlines operations and increases productivity without processing delays. It has the function of possible future analysis and generates reports as per customer requirements and for their business deployment and improvement ideas [35].

Data integration is a cost-effective and time-saving tool. It provides automation and analysis of application and connected server data flow. It increases the productivity and efficiency of the process and reduces the number of errors [36].

However, since there is a probability of data loss or data mismatch when extracting and filtering data from different sources, a data integration system minimizes all these impacts, as it provides automatic data sharing between client and server. It can be easily updated and synchronized at any time as an instantaneous process [37].

It is a centralized system that provides many quality services to different domains connected to a main network. The accuracy and reliability of data is thus maintained throughout the network. Popular organizations such as Google or Facebook process information about the inflow of transactions with billions of people in every corner of the world within milliseconds [38].

The magnitude of information generated is considered big data. Often, businesses that work with data warehouses can focus on big data. All the data collected becomes significant to the enterprise, which means that the analysis that can be performed in terms of implementation is easier. Big data are good for creating analytics solutions. Integrated data are essential for processing and evaluation. Big data have distinct characteristics that differentiate them from statistical enterprise data. Traditional data warehouses and data management tools are not ready to process and analyze large volumes of data in a very short time (sometimes in real time) or in a cost-effective manner. Therefore, there is a need to find new ways to process and analyze big data [39].

### 2.5. Data Integration Requirements

The key data integration requirements are simplified in the following sections:

- It must be able to integrate any data from any source;
- Data should be stored either in the cloud or on local storage;
- Maximum performance should be provided;
- Provides broad-based support and trusted information;
- Providing data on the quality of the production processes;
- Insight into the quantity and quality of products.

With new trends in the industry, data integration is more important than ever. Businesses today face challenges when dealing with large amounts of data. They should use more powerful and flexible data integration technologies to transform data into a usable form. All of the new requirements for data integration have led to the development of a new area of integration referred to as the integration platform as a service [40].

### 2.6. Overview of Data Produced at Heterogeneous Levels of Governance

Table 1 shows a basic overview of the data that can be produced at different levels of management and their use in improving the production process.

As shown in Table 1, intelligent peripherals, actuators, and sensors are used at the direct control level. Systems that previously operated in isolation are connected to them via industrial buses. 

Most often these are programmable logic controllers (PLC) or industrial personal computers (IPC). The supervisory control level is represented, for example, by human machine interface (HMI) panels used for real-time data monitoring. At the production resource planning level are information systems that integrate more powerful computers connected to the enterprise information system. Together, these two levels constitute information and control systems [45].

### 2.7. General Models of Integration

Two basic integration models are used in practice to implement the interconnection of systems and application data from multiple levels of management. The first model represents an object-oriented integration design. The second model is represented on the basis of TAGs, which represent the logical encapsulation of controls, systems or applications, or even any data source.

#### 2.7.1. Object-Oriented Integration Method

This method is based on defining inter-relationships. Thankfully, in objects, details are easier to see. Less important or uninteresting facts can be omitted from the definition as part of the abstraction, thus simplifying the implementation of the integration. This model is commonly used for the implementation of control systems or data integration. Its disadvantage is that the visual aspect of the integration is, in most cases, implemented last. For SCADA or MES control, a graphical representation is preferred. However, integration implemented in this way, by using object-oriented development, can save technical resources considerably. 

For an object-oriented integration model, an abstract model needs to be defined so that the developer can realize the implementation. In industry, it is necessary to perform analysis and abstraction of the layout of production cells to perform an identification of the processes that are part of the production and to create a model of their communication or dependency on each other. Alternatively, if the communication cannot be identified, at least their interconnection in terms of data representing the potential interconnection of the individual cells of the model must be identified [46]. 

**Figure 2:**
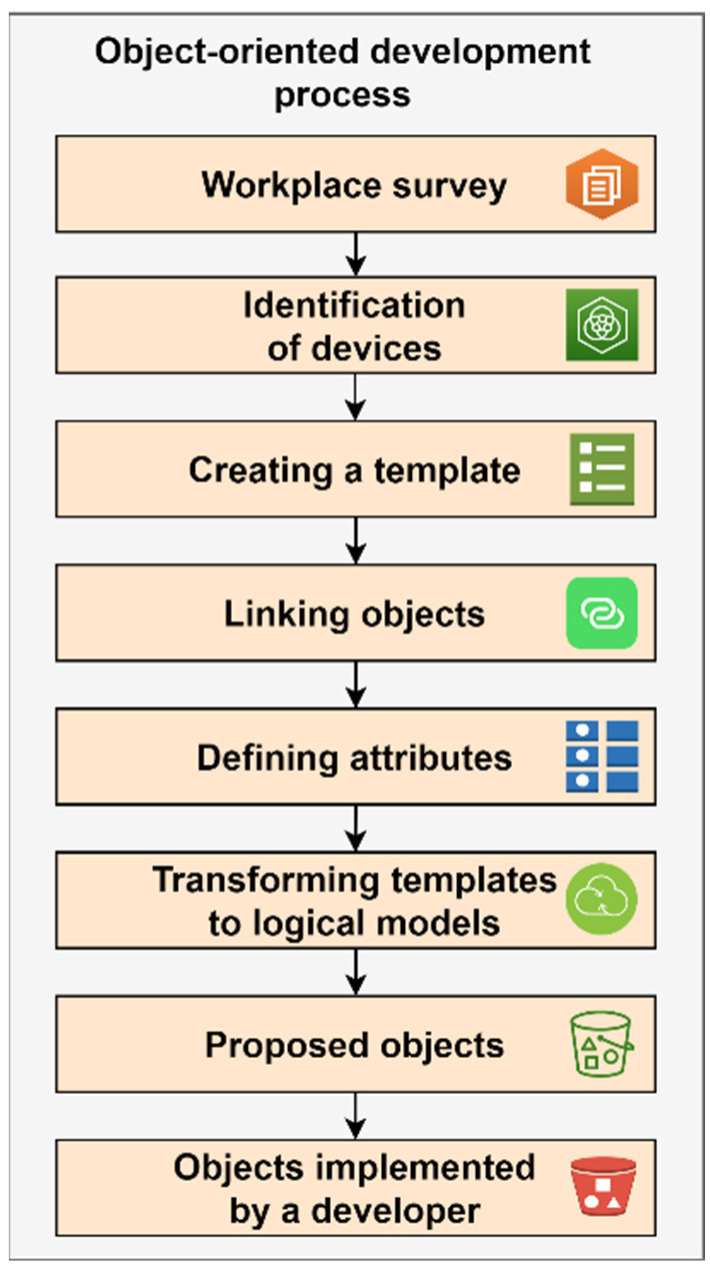
Object-oriented integration model.

The procedure for creating the object-oriented integration model is shown in Figure 2.

1. A site survey shall be performed to understand the layout of the manufacturing operation or process. Nonstandard or specific use of technology shall be defined.
2. A list of similar equipment shall be developed. There are common types of motors, valves, transmitters, control loops, actuators, etc., identified.
3. Templates for similar or compatible equipment or components shall be configured or created. In this way, common standards will be set for applications that will communicate or monitor processes or controls. The prepared templates can be used to develop objects representing a specific device.
4. Device templates can represent classical object associations and they can be represented by inheritance relationships. One object abstraction can contain another to create more complex devices. In this way, a compatible design for object-oriented programming is guaranteed.
5. The device templates have defined attributes. These represent the actual inputs/outputs available in the control system. For PLCs, these can also be defined as address spaces that contain the values required for process control. This activity results in attributes mapped to input/output (I/O) elements via device integration objects.
6. When templates are transformed into logical device models, they can be transformed into object instances. This creates an object that is mapped to the template. It provides the basis for implementation and should be well enough specified to allow additional changes and new requirements to be applied to the system if necessary.
7. The designed objects should be classified as security groups. This can be performed individually as needed based on enterprise definitions. Security groups must contain common attributes and security permissions. Roles are created for mapping and, in this way, general data protection regulation (GDPR) can be applied.
8. Objects implemented by the developer are then implemented and usually run on application servers. Usually, the design is implemented as a distributed system that can reside on a single server or run on dozens of servers that are clustered together, and, this way, stable operation is ensured even during hardware failures. This design is referred to as redundant resource provisioning or fail over cluster.

The advantage of the object-oriented design is the simple and standard representation that is applied to the implementation of software development; it is possible to achieve the implementation and deployment of integration in this way using resources that are not specialized for deployment in the industry. Applications and development environments are not limited elements and their realization and implementation can be accomplished without the in-depth knowledge of the programmer, who does not need to be a specialist oriented to manufacturing technologies and equipment. The disadvantage is that it can be longer and more difficult to implement.

#### 2.7.2. TAG-Based Integration

Software development in this industry has had several stages. This type of integration is based on the need to visualize the integration of processes for the operators to ensure easy extensibility but also to ensure operator control of the production process. The visual realization of the integration and interconnection of the control elements is usually available to the production operators on HMI panels, in MES/manufacturing operations systems (MOSs) or SCADA systems. This principle is based on the necessity of creating graphically unrepresentable interconnections. To fulfill this goal, abstractions of signals, data, and activities that occur in the control systems of production lines have been created and are represented by TAGs—tags representing addresses in PLCs and control systems—or are data or events that arise during production control. 

For this type of integration, it is necessary to ensure that the system is compatible with the supplied control application. Typically, the MES/MOS supports integration using TAGs and its modules ensure compatibility so that each control member is represented by a module that ensures its integration. This means that a TAG can be defined for a compatible device supported by the system, where the module will ensure communication with the device. Simultaneously, this module maps the attributes, data, and signals and represents the control member of the production line using a logical representation in a definition denoted as TAG (tag) [47].

**Figure 3:**
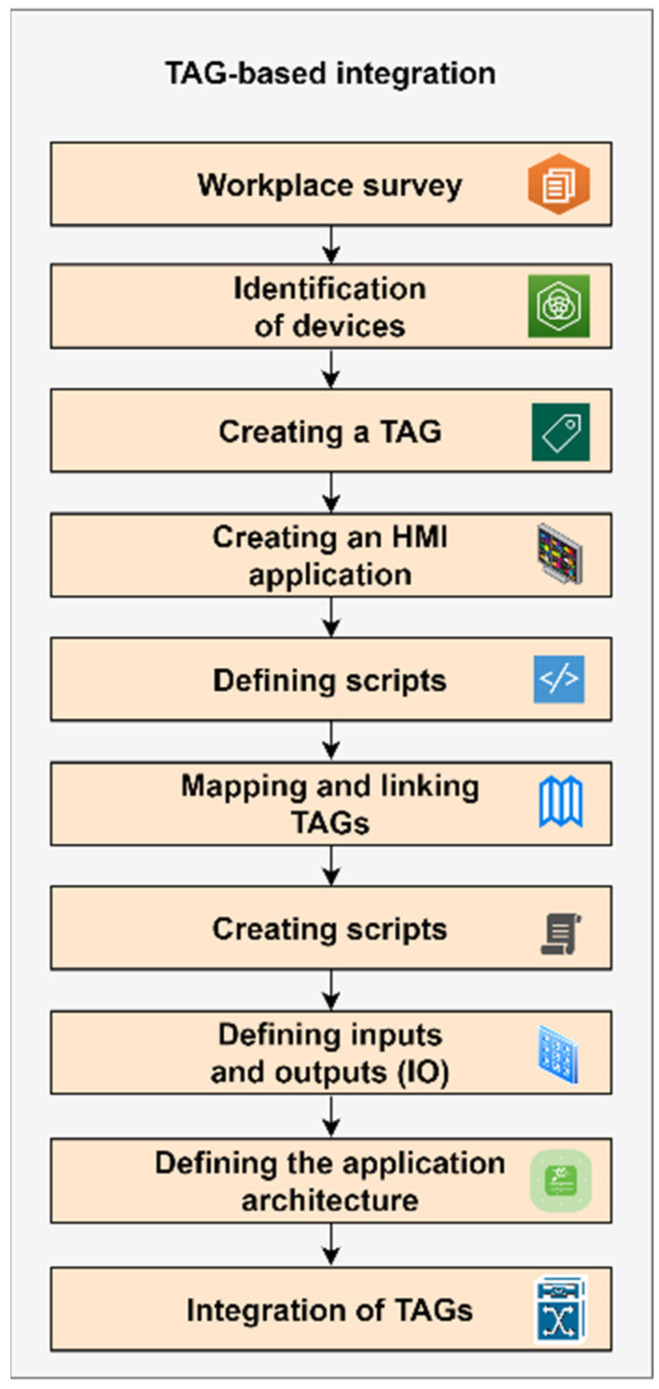
TAG-based integration model.

TAG-based integration, shown in Figure 3.

1. A site survey shall be performed to understand the layout of the manufacturing operation or process. Nonstandard or specifically used technologies shall be defined.
2. A list of similar equipment shall be drawn up. There are common types of motors, valves, transmitters, control loops, actuators, etc., identified.
3. A tag (TAG) is created for each type of device or control element. Data, events, or attributes are automatically imported from the PLC or manually configured according to the type of control element.
4. To integrate the TAGs, a new HMI application is created for which windows or displays are created.
5. Scripts are defined for each TAG to detect alarms and events.
6. Tags are mapped and linked to the graphical elements of the HMI applications.
7. Scripts for graphical animations or links are created.
8. IO tags are defined, if necessary, and interfaced with the application.
9. If the application is to be deployed in a client-server environment, the application architecture is defined to centralize alarms and event detection, and perform data archiving.
10. When integrating using TAGs, each change in the system is applied by turning the application off and on. This is due to the application and execution of script changes, and due to references to the TAG database to enable new functionality and reload the new HMI on each workstation.

TAG-based integration allows one to maintain a significant portion of existing automation and information infrastructure and to integrate and synchronize existing production systems and new applications. Since this is a higher-level integration, mapping existing resources to TAGs and integrating them can represent a reduction in project lead time and overall development costs. An important prerequisite is the correct selection of the application that will be used to implement the integration using TAGs. It is necessary to identify and guarantee compatibility with the controllers and to verify the possibilities of extending the integration with the protocols that are supported.

## 3. Results

There are several fundamental differences between object-oriented integration design and TAG-based design. Table 2 describes the basic differences in the way that object-oriented versus TAG-based integration processes differ.

Manufacturing companies are investing heavily in data collection and analysis and in the infrastructure of their production lines. However, many of the control systems may not support Industry 4.0 technologies. Therefore, it would seem that legacy equipment leaves a gap between the collection of production data and the control capabilities that can be performed with them, but even the legacy equipment allows for a level of integration that, while it may be limited, is usually, at least, partially possible.

### 3.1. Basic Data Integration Model

The basic data integration model consists of the following stages.

- Design. In the design phase, first, it is necessary to determine what the objectives of the data integration are and to identify the sources from which the data will be obtained. Other attributes that need to be considered in the design of the data integration are data availability, data retrieval capabilities of the data sources, quality level of the retrieved data, backup capabilities, and security.
- Implementation. Based on the requirements analysis and the SRS (software requirements specification) document, suitable tools are selected for the implementation of the data integration system. In the case of companies that do not have an integration system, a new tool must be implemented, and companies that have implemented or are using the implementation can only extend their system based on the requirements that arise.
- Testing. The testing phase is critical because it is necessary to ensure that the data are unified, accurate, and correct. Different testing methods can be used, such as the performance stress test (PST), technical acceptance testing (TAT), and user acceptance testing (UAT).

**Figure 4:**
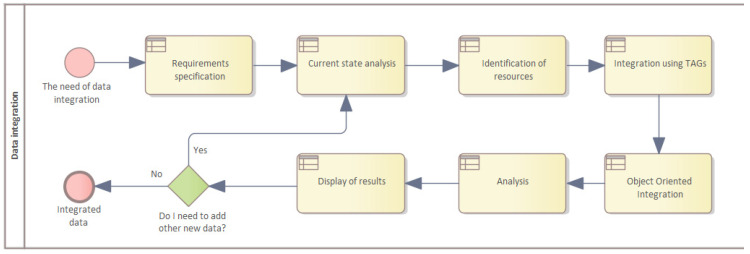
Data integration model.

The data integration process consists of several steps, as shown in Figure 4. If a manufacturing company decides that it wants to integrate the control of its manufacturing process, the first step is to specify the requirements, what the integration should meet, and what the goals and benefits will be. The next step(s) to be undertaken is the current state analysis, i.e., a description of the current situation and how the enterprise operates before integration. Subsequently, it is necessary to identify all available and essential sources from which data will be integrated and then process this data into a suitable form for storage in data warehouses, as required.

Data processed and stored, in this way, can then be analyzed and the results evaluated, which can lead to either the identification of deficiencies during the production process, i.e., production efficiency, or cost reduction. If not, all of the data required are integrated and the integration process moves again to the current state analysis phase.

In the actual creation of the model, it is necessary to ensure that the signals are defined. Signals are obtained from the managing members. To establish communication between the production line stations and the system platform management console (SMC), it is necessary to establish a connection with the individual controllers, IoT devices, and PLCs at the stations. This connection is created by adding the individual IP addresses of the devices and then configuring them according to the production line documentation. 

**Figure 5:**
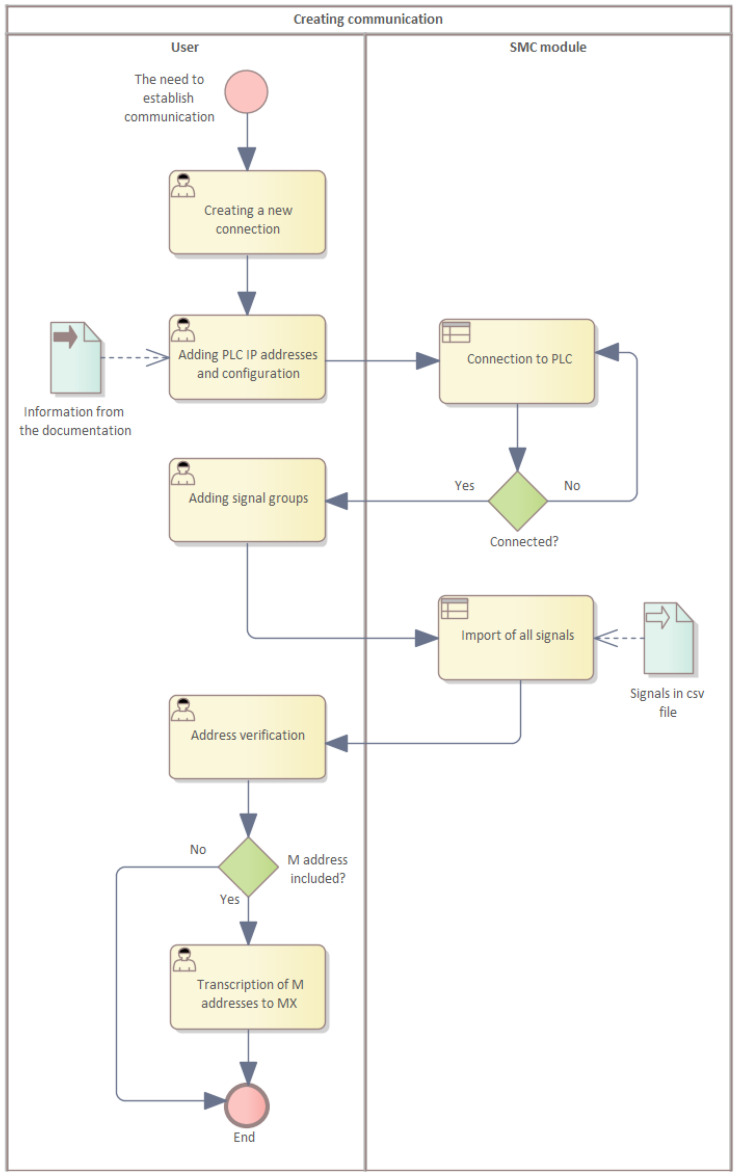
Establishing a connection between the PLC and the SMC.

Once this connection is established, it is then necessary to define the individual signal addresses that the stations contain. These are usually available from the documentation or a prepared comma-separated values (CSV) file in the required format. The CSV file has been prepared on the basis of the documentation supplied for the individual control elements of the production line. It contains the address spaces of the PLC devices, their description, the communication method, the interface type, and error codes. This process is represented by the business process diagram shown in Figure 5.

In addition to the classical inputs and outputs, memory variables, commonly denoted by the letter M and their absolute address, must be imported. The only change that needs to take place is that one must rewrite the signals with address M to indicate address MX. This step depends on the system used. Sometimes, mapping of signals by reference is not required. If necessary, a physical mapping of M to logical MX must be created by the system.

After the connection is established by adding the IP addresses of the PLCs to the SMC module, it is also important to establish communication in the MES by creating individual top-levels and importing the signals from the CSV file. Here, it is necessary to create individual “topics” for each station and for each group of signals. Then, the individual signals must be imported into these groups.

**Figure 6:**
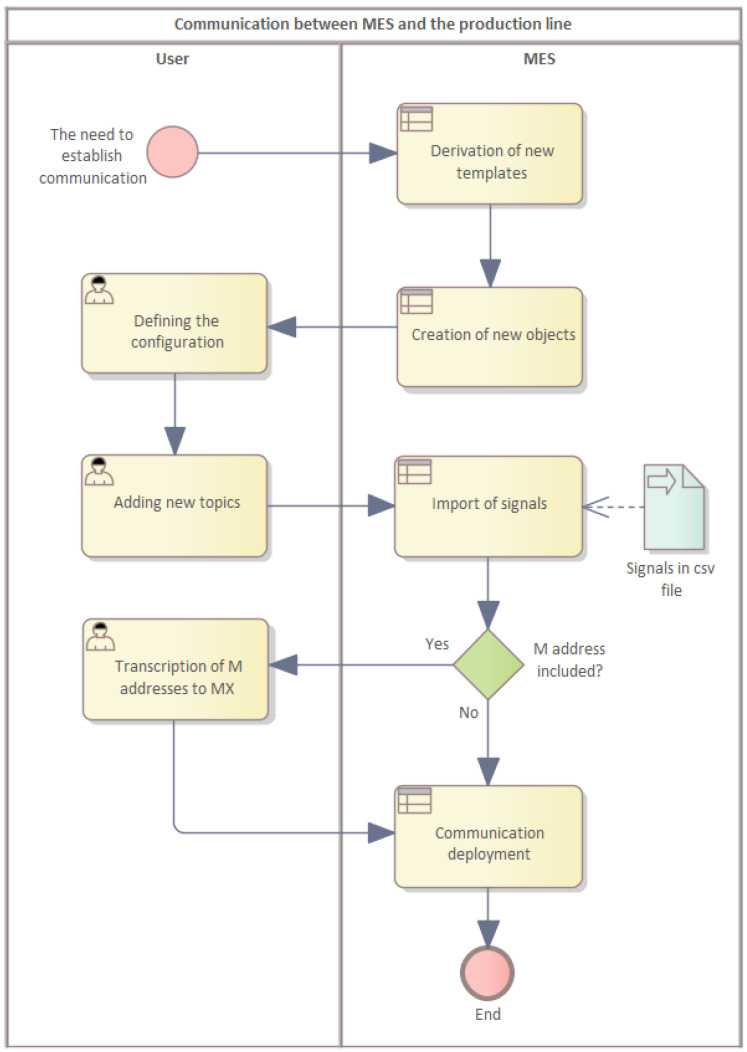
Communication between the MES and production line.

The communication was established via MES, where it is necessary to first connect the individual PLC devices by adding individual IP addresses to the SMC module and then configuring them as shown in Figure 6.

The modules need to be selected to include a supported hardware version or universal industrial protocols. This depends on the MES software installed.

### 3.2. Generalization of the Design to Use Integration in an Industrial Enterprise

**Figure 7:**
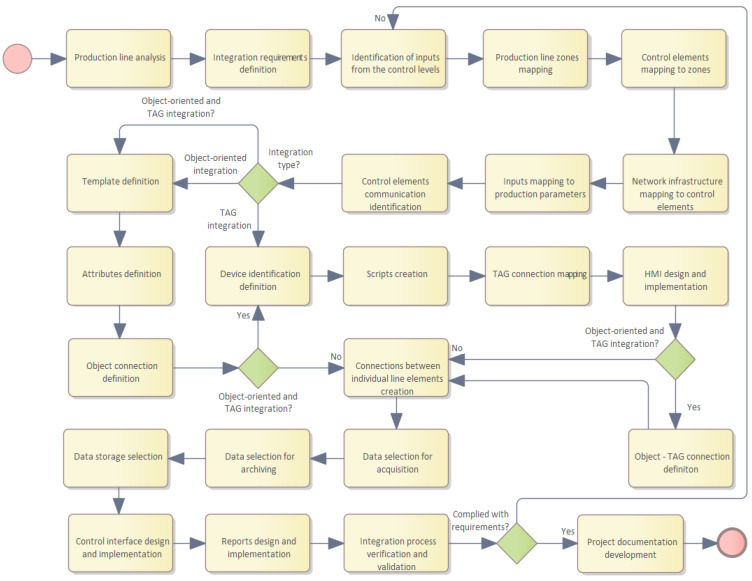
General proposal for an industrial integration process.

The creation of the integration of the proposed procedures using the object-oriented model and the integration using TAGs on the production line demonstrates the applicability for the general application of the given procedure for integration in manufacturing enterprises, which is shown in Figure 7.

An analysis of the production line must take place so that a deconstruction of the whole into elementary parts can be carried out. In the analysis, it is necessary to determine these elementary parts and their relationships, to determine the essential parts, and to define their importance and regularities. The result of the analysis is the formulation of the problem, which serves as the basis for the creation and definition of the integration requirements. 

Defining the integration requirements and determining the service-level agreement (SLA) also determines strategic requirements such as data management, accessible and open data, data importance and parameterization, data management implementation, and interoperability within the intended integration.

This is followed by the identification of inputs that come into consideration in the integration from all levels of management, and includes all types of data, including documents, controls, and documentation. Inputs are representatives of potential data with different data types and formats. It may also be possible to determine whether data transformation or format validation needs to be provided for an identified input or not.

Mapping production line zones represents the creation of a logical and physical arrangement of production zones. Mapping is a process that is complemented by a description of the functionality of the production zone. The activities, equipment, and control elements that are used in the production process are identified. 

Control mapping is a process in which the types of control, the technical specification of the controls, the communication options, and the available interfaces that can be used in the integration solution are identified. Controls should be categorized into groups that have similar characteristics or classified according to compatibility or control type.

The network infrastructure must be mapped to the control elements. This section provides a diagram of the network infrastructure, the network components used, and their mapping to the physical infrastructure elements. At the application level, the identification of addressing and assignment of physical ports of the control members and network communication devices is performed. 

Mapping inputs to production parameters is a process in which it is necessary to determine which inputs will be applied to control and influence the production process. Inputs to production, once abstracted, represent data that specify parameters that affect the production process. Determining the data from each input and assigning them to integration is necessary for designing the integration solution.

The identification of the communication of the control elements determines the type of communication and protocol, or the method for addressing the control elements. According to the technology used, the data and their priority are parameterized and determined. In control, it is necessary to identify the types of protocols that are used for control and represent real-time data transmission. For these, it is advisable to verify whether integration is possible or whether the modification of the existing setup is necessary so that possible integration does not compromise the reliability and speed of information transfer.

Based on the steps performed so far, it is now necessary to determine which integration method is suitable for the task at hand. The characteristics of the solutions offered must be considered. As a rule of thumb, if the task is simple, unstructured, and visualization is needed, TAG integration is applied. If the integration is more complex, structured, has defined dependencies, and does not need visualization, the object model of integration is appropriate. In the case of both requirements, a combination of both can be applied by first preparing object type integration over which a TAG extension is built. It is valid that the object implementation represents a logical device for the TAG integration model.

For object–type integration, the definition of the templates must be followed. This represents an abstraction of the problem to be solved. A template can have common attributes and these can be modified. A template can be made into a new template by inheritance and the required parameters or attributes can be modified.

Attribute definition is the step where a list of attributes is created. Each attribute has specified parameters, limits, and properties or constraints within which it can receive values. An attribute represents a property that is available in the control process and can change the behavior of the created object.

The definition of object links is the creation of object links that provide for interaction and communication between objects.

Creating links between line elements is again the creation of a link map of the line control elements, where the logical continuity of the control data exchange is provided, and by using this step, it is possible to determine how the line control elements interact with each other. 

In the TAG integration model, a definition for the identification of the devices is necessary. The control device is identified by technical and functional parameters. An abstraction of its attributes is created and a mapping of the physical interrogation is created so that its attributes, inputs and outputs, memory addresses, etc., are available for software implementation. By defining a TAG, a logical model of the device is created that can be addressed.

Creating scripts is the step where the addition of security functionality occurs. Scripts for alarm and event detection and scripts for graphical animation or links are defined.

The design and implementation of the HMI include a graphical implementation that visualizes the integration and provides possible interaction of the production operator with the control process. The visualization part of the HMI implementation is simpler for the so-called flat type of tasks, without complied branching and dependencies, and the TAG type of integration is suitable for these types of tasks.

TAG link mapping is the creation of integration based on TAGs to ensure their continuity and connection. Each TAG can be logically linked to another TAG, and data exchange between them is possible.

Design and implementation is the last step in TAG integration, where the actual implementation of the integration can proceed based on the previous steps.

Defining object-TAG links provides a combination of both types and is the most commonly encountered type of integration. It is a combination of the advantages of both approaches and, therefore, this solution is usually applied in such a way that the basic functionality is solved as object–type integration and the visualization is realized using the TAG approach. Object-tag interfacing is implemented by mapping an existing object containing the implemented functionality to a virtual or logical TAG. Thus, it is available for implementation in TAG integration.

The selection of data for the collection consists of the decision to determine which data need to be recorded. This activity is closely related to the objects and TAGs created, as the selection of the available data contained using the given integration procedures is then performed. The data were recorded in an unmodified state. If the modification is required, it is recommended to create a transformation of the original value and store the newly created data under the new identifier.

The selection of data for archiving is necessary for the definition of archiving policies and security rules or compliance with regulations and laws such as GDPR. They are used for long-term archiving. They are data that have a certain level of importance and are significant from a business management perspective. Other types of data may be involved, but these are generally determined by the needs of the entity. The archival volume of data should be smaller than the data collected. It is also common in this section to archive data that are collected from multiple levels of management, including at the document, file, etc., level.

The choice of data storage is a task that determines how the data will be stored and what system will be used for data storage. Depending on the type of data, these systems may differ and have different requirements. For integration data, is usually file storage, for sequential data storage, a relational database is usually used, and for a combination of both, a module or application is usually available to provide comprehensive access to both the aforementioned types of data.

The design and implementation of the management interface is the actual implementation of the integration and combines all the previous steps into a comprehensive integration solution. The implementation is the creation of the application, the control systems, and the realized intent of the solution. In integration software, this part is responsible for the control, but also the visualization of the production process and, thus, enables the implementation of the integration.

The design and implementation of reports represent the design of reports and tools that enable decision making in the management process. These reports use integrated data, where they are aggregated, analyzed, and eventually transformed into documents, reports, and statistical summaries that can be used in the management of the company. 

Verification and validation of the integration process is the last task, which is a standard step in the deployment of integration or software in general. Verification is used to check the correctness of the product against the planned requirements of the integration solution. Validation, in turn, addresses the verification of the correctness of the integration with respect to the real requirements.

Documentation creation is the step where all the essential implemented steps, their parameters, created connections, maps, object definition, TAG attributes, schemas, and network parameters are recorded in the technical documentation. 

The documentation can be categorized by activity type or technical focus. It serves as the output of the implementation and is the basis for any further modifications and changes, where each version is documented and the changes made with respect to the previous version are recorded.

## 4. Discussion

**Figure 8:**
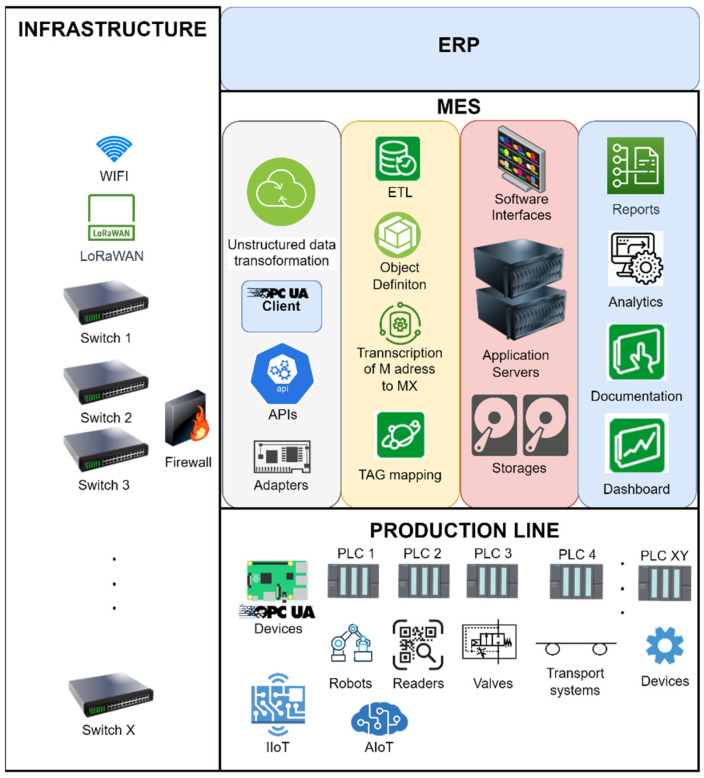
Data integration from heterogeneous control levels.

Data integration, which uses the selected models, was finalized by designing and creating a concrete TAG mapping implementation and defining object templates. Based on these, it was possible to derive the necessary objects and their attributes. This increased the possibility of a visualization of the manufacturing process where, when describing the elements in the MES system, it is possible to assign a representational visualization form to these elements. The creation of the visualization can be used directly by the defined properties of a given TAG or object. Data integration from heterogeneous control levels is shown in Figure 8.

The advantage of the solution is a simple and a fast possibility of realizing integration from heterogeneous data sources. The described procedures represent a universal integration solution that covers various applications, not only in industrial environments. Both mentioned types of models are extensible and applicable according to the suitability and evaluation of the complexity and structuring of the task. Both represent a suitable type of solution and deployment in practice.

The disadvantage of the solution is the dependence on the choice of the delivered system and the possible assurance of its compatibility with the used systems and components that need to be integrated. In this area, adherence to Industry 4.0 standards seems to be a potential solution, and for the specific application of industrial equipment, the modern open solution OPC UA is quite well supported. However, in this case, it is also true that there are multiple versions where especially older devices or software implementations may not support the latest type of communication. 

In the case of OPC UA deployment, it is necessary to verify its older version, generally referred to as OPC DA, which is, however, limited to data access only by its name, DA—data access. 

In a similar proposal, described by R. Beregi et al. [48], a basic methodology was presented to universally model, digitize, and integrate the services offered by different isolated work cells into a single, standardized and extended production system. This methodology is bound for the industrial layer that uses OPC UA. In this article, a model is presented that can be applied to different control layers.

Gradual implementation has resulted in an integration that supports HMI visualization suitable for operators, but also provides high-value views for management that cover areas ranging from production quality, evaluation of production flow and times, production defect rates, and quantitative evaluation of production operations and their statistical evaluation. 

It is possible to track the movement of a product from order placement to finalization. In the case of a situation where a production management problem is identified and the analysis reveals that the required new data are not available in the existing technical means, the integration of additional equipment comes into consideration. However, the addition of data and control signals allows the production process to be changed without the need for changes to the original controls with minimal risk.

The offered procedures and the presented integration models represent a modern, reliable, compatible, and widely applicable general solution for industrial and other manufacturing companies.

## 5. Conclusions

In this article, the analysis of the current state of the problem area of integration of heterogeneous enterprise data from different levels of management and the creation of an overview of the theoretical background and knowledge in the field of the issue was carried out. On the basis of the developed analysis, it was possible to establish the objectives and determine the direction of this dissertation. 

Thanks to the elaboration of the analysis and overview, it was possible to propose solutions for enterprise data integration. Two basic integration models are proposed in this thesis. 

The first is the object-oriented approach, which is suitable for solving complex and structured tasks. The second type of model is the TAG approach, which is suitable for solving tasks that are less complex, require a graphical representation, and are designed for flat-type tasks that do not require complicated dependencies or complex interconnections. 

The two types can overlap, and the most common method of integration in real-world environments is to combine them, where the object model is responsible for the more complex tasks, and the link to the TAG model is implemented by a logical mapping of the object to the TAG. 

This provides a link and ensures the advantageous functionality of both models. Both models are analyzed in detail and recommendations are made on the steps that need to be implemented to ensure that their design is appropriate for the types of tasks for which they are intended. 

The object-oriented approach represents a basic approach that is suitable for complex and structured solution designs. The TAG-based approach is designed for less complex integration implementations where visualization or interaction using graphical HMI interfaces is desired. Based on the theoretical and practical experience of production line integration using object- and TAG-based models to support simulations, a generalized integration model was proposed that applies to the design and implementation of enterprise integration.

The developed generic model, which considers both approaches, is suitable for the implementation of integration in the industry. The model is broadly applicable and considers data sources from different levels of management. According to the developed model, the data to be integrated can be processed as needed in the company’s management systems. Thus, it represents the basis for the implementation and deployment of an MES and the creation of a basic concept for the integration design and implementation of an industrial production line. It can serve as a basis for further research in the field of integration in industry.

## Figures and Tables

**Table 1:** An overview of the data produced at the heterogeneous management levels.

Management Level	Data Type	Purpose of Use
Direct control [41]	Data from specific devices and sensors (PLCs, IIoT devices, controllers)	Real-time control of specific devices
Supervisory control and SCADA and HMI data collection [42]	Visualized data from the HMIs	Immediate monitoring and production control
MES production control and tracking [43]	Detailed production tracking data	Use for business and management purposes
MRP/ERP production resource planning [44]	Data from order processing, inventory management, financial modules, and production planning	Management of the organization as a whole (logistics, finance, etc.)

**Table 2:** Comparison of object-oriented and TAG integration.

Object-Oriented Integration	Integration with TAGs
Tasks that need to be structured and have dependencies created	Simple tasks, controls that do not require structure and hierarchical links
Providing inheritance or compatibility for devices that may differ in a few small attributes, or share many common characteristics, and can be templated well	Variable control elements that do not have a common attributes. It makes little sense to create a template for them
A large number of parameters need to be monitored	A large or small number of monitored parameters
Unnecessary visualization	The need to create a visualization of the components on the HMIs
High functionality in terms of application logic	Low complexity for implementing application logic
Potentially frequent functionality changes	Low probability of functionality change

## Data Availability

Not applicable.
